# Enlarged Cavum Septum Pellucidum as a Neurodevelopmental Marker in Adolescent-Onset Opiate Dependence

**DOI:** 10.1371/journal.pone.0078590

**Published:** 2013-10-24

**Authors:** Jaeuk Hwang, Jieun E. Kim, Marc J. Kaufman, Perry F. Renshaw, Sujung Yoon, Deborah A. Yurgelun-Todd, Yera Choi, Chansoo Jun, In Kyoon Lyoo

**Affiliations:** 1 Department of Psychiatry, Soonchunhyang University College of Medicine, Seoul, South Korea; 2 Department of Brain and Cognitive Sciences, Ewha Woman's University Graduate School, Seoul, South Korea; 3 McLean Imaging Center, McLean Hospital, Belmont, Massachusetts, United States of America; 4 Department of Psychiatry and The Brain Institute, The University of Utah, Salt Lake City, Utah, United States of America; 5 Department of Psychiatry, Catholic University of Korea College of Medicine, Seoul, South Korea; 6 Interdisciplinary Program in Brain Science, Seoul National University College of Natural Sciences, Seoul, South Korea; 7 Ewha Brain Institute & College of Pharmacy, Graduate School of Pharmaceutical Sciences, Ewha Woman's University, Seoul, South Korea; King's College London, United Kingdom

## Abstract

**Objective:**

Adolescent-onset exposure to highly addictive substances such as opiates may induce far-reaching deleterious effects on later mental and physical health. However, little is known about the neurodevelopmental basis for adolescent-onset opiate dependence. Here we examined whether having an abnormally large cavum septum pellucidum (CSP), a putative marker of limbic structural maldevelopment, is associated with opiate dependence particularly beginning in adolescence.

**Method:**

The overall length of the CSP and the prevalence of abnormal enlargement of the CSP were assessed and compared in 65 opiate-dependent subjects (41 adolescent-onset opiate users and 24 adult-onset opiate users) and 67 healthy subjects.

**Results:**

Opiate-dependent subjects showed a greater prevalence of abnormal CSP enlargement relative to healthy subjects (odds ratio [OR]=3.64, p=0.034). The overall CSP length of adolescent-onset opiate-dependent subjects was greater, as compared not only with healthy subjects (F_1,104_=11.03, p=0.001) but also with those who began opiate use during adulthood (F_1,61_=4.43, p=0.039).

**Conclusions:**

The current findings provide the first evidence that abnormal CSP enlargement, which reflects limbic system dysgenesis of neurodevelopmental origin, may be linked to later development of opiate dependence. In addition, a greater CSP length, which indicates more severe limbic abnormalities, appears to confer higher risk for earlier onset of opiate use.

## Introduction

An earlier onset of substance abuse particularly during adolescence has been suggested to be related to chronic relapsing medical course in adulthood [1,2]. As the long-lasting implications of adolescent-onset substance abuse draw more attention, researchers also seek potential neurobiological markers that may identify adolescent subgroups with high vulnerability to experimental use of illicit drugs and subsequent development of dependence on these drugs.

As one candidate of the primary neuroanatomical correlates of substance dependence, the limbic system has received much attention [3,4]. The limbic system, together with the prefrontal cortex, plays essential roles in mediating emotional and motivational behaviors including rewarding-related behaviors in substance abuse [3]. Indeed, a growing number of neuroimaging studies have reported a range of functional and structural abnormalities of the limbic system in substance-dependent subjects [4].

Although it is yet unclear whether limbic abnormalities might predispose or be secondary to substance dependence, a recent study of high-risk individuals for alcohol abuse presented evidence in favor of a neurodevelopmental role of reduced amygdala volume in later development of alcoholism [5]. Furthermore, it has also been suggested by a recent review that immature prefrontal control of the limbic system in the adolescent brain may account for the developmental vulnerability to addiction in teenagers [6]. Consistent with these perspectives, fetal maldevelopment of the limbic system has been hypothesized to be a neural correlate of increased risk-taking and sensation-seeking behaviors [7]. Taken together, pre-existing brain-based susceptibility might, in part, contribute to the development of earlier risk-taking behaviors such as illicit drug use as well as full-blown addictive behaviors [8].

There have been, however, few reports regarding whether and how premorbid regional abnormalities of the limbic structures play a role in the development and progression of substance dependence [8,9]. Furthermore, inter-individual brain regional variance to predict an earlier onset of illicit drug use remains as an important issue to be resolved. Longitudinal human studies could help to disentangle the pre-existing neurodevelopmental vulnerabilities to the neuroadaptive/neurodegenerative changes of limbic structures regarding substance dependence. However, this approach may not be feasible due to the difficulties of conducting research with cohorts in which most will not be developing that particular disorder. However, efforts to assess anatomical markers for the neurodevelopmental abnormalities of limbic structures [10-12] might be of value both for elucidating pathophysiological roles of these brain regions in substance dependence and for helping to identify at-risk individuals to enroll into longitudinal studies.

In normal brain development, the leaves of the septum pellucidum fuse as a result of the continued development and growth of the corpus callosum as well as limbic structures, including the hippocampus, the amygdala, and septal areas, within 3 to 6 months after birth [12]. Incomplete fusion and subsequent occurrence of the cavum septum pellucidum (CSP), a space or cavity between the two leaves of the septum pellucidum, has thus been recognized as a neurodevelopmental marker of limbic system dysgenesis, particularly when the size of CSP is unusually large [12]. In this regard, the presence of an enlarged CSP has been as a marker of predisposing neurodevelopmental abnormalities of the limbic system in several psychiatric disorders. For example, an abnormally large CSP has frequently been observed in patients with schizophrenia or affective disorders, all of which have neurodevelopmental models as etiological hypotheses [13-17]. However, to the best of our knowledge, early neurodevelopmental lesions such as an enlarged CSP have not yet been examined in substance dependence.

In this study, we sought to investigate the prevalence of an enlarged CSP in a large sample of opiate-dependent subjects and carefully matched healthy subjects. Based on the explicit temporal causality between the occurrence of CSP and the onset of opiate dependence, we hypothesized that individuals with an enlarged CSP would be more prone to have opiate dependence than those without. Furthermore, we expected that a larger CSP would be associated with an earlier onset of opiate use potentially due to a greater neurodevelopmental disability of the limbic system during adolescence, when prefrontal cortical control has not fully matured.

## Methods

### Ethics statement

Institutional Review Boards of Massachusetts General Hospital, McLean Hospital, Boston Medical Center, the Boston VA Healthcare Systems, Seoul National University, and Ewha Woman's University reviewed and approved the research protocols. Written informed consent was obtained from all participants following a complete explanation of the study procedures.

### Participants

In the present study, a total of 65 individuals who were diagnosed as having opiate dependence using the Structured Clinical Interview for DSM-IV (SCID)[18] and 67 age- and sex- matched healthy subjects were included. All participants were between 18 and 60 years of age. The current sample overlaps in part with that of previously published study described elsewhere [19].

The presence of current abuse/dependence on psychoactive drugs other than opiates, alcohol, or nicotine was an exclusion criterion. Subjects with concurrent major psychiatric, neurological, or medical disease, a history of head trauma with loss of consciousness, or any contraindications to magnetic resonance imaging (MRI) were also excluded from the study. The same exclusion criteria, except for a diagnosis of opiate or alcohol abuse/dependence, were applied to healthy comparison subjects.

In order to examine the effects of limbic developmental abnormalities on the onset of opiate use, the opiate-dependent subjects were divided into two subgroups, individuals who began opiate use before age 21 (n=41, hereafter defined as the 'adolescent-onset opiate-dependent group') and those who began after age 21(n=24, hereafter defined as the 'adult-onset opiate-dependent group'). 

### MR image acquisition and image processing

MR image data from each participant were obtained using a 1.5 tesla General Electric Signa scanner (Horizon Echo-Speed, General Electric Medical Systems, Milwaukee, WI, USA). We used a custom-made receive-only linear birdcage coil which improved signal-to-noise ratio and homogeneity by approximately 40% of rate as compared with standard quadrature head coils. One hundred twenty-four contiguous T1-weighted 1.5-mm-thick coronal images were produced using a three-dimensional spoiled-gradient echo pulse sequence (echo time=5 ms, repetition time=35 ms, 256 x 192 matrix, field of view=24 cm, flip angle=45 degree, 1 number of excitation). T2-weighted images and axial proton-density images were also acquired for structural abnormality screening.

Acquired T1-weighted images were transformed into a single Analyze format image. Then, voxels of each image were interpolated to 0.5 mm^3^ and the images were realigned spatially to the anterior commissure-posterior commissure plane using Analyze 9.0 (Mayo Clinic, Rochester, MN, USA). All images were examined for gross structural abnormalities and developmental anomalies and rated for image quality by a board-certified neuroradiologist who did not know subjects' diagnosis or clinical information.

### Measurement of CSP

**Figure 1:**
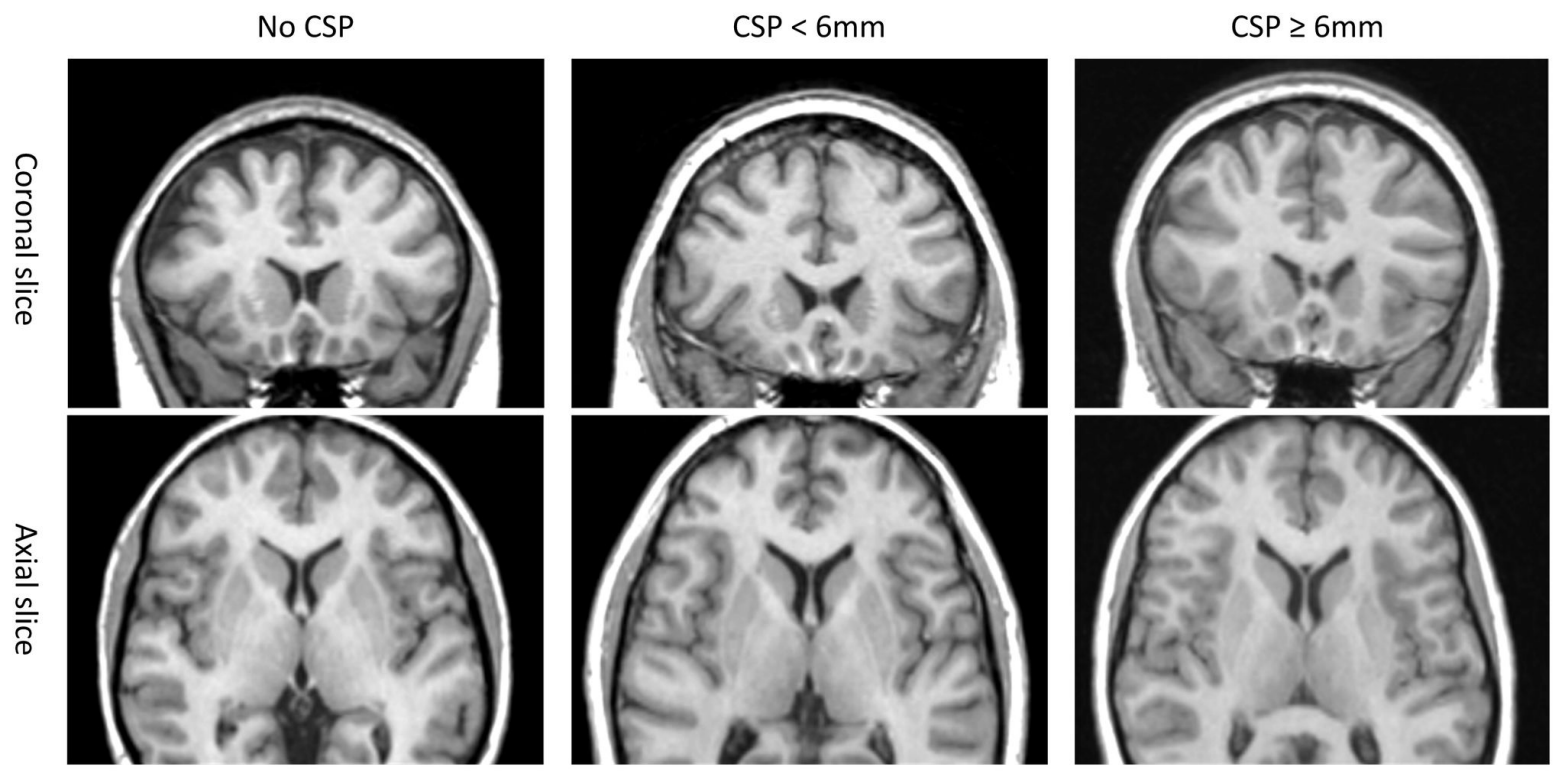
Illustration of normal septum pellucidum and cavum septi pellucidi ranging from small (< 6mm) to abnormally enlarged (≥ 6mm) in opiate-dependent subjects. Abbreviations: CSP, cavum septum pellucidum.

An experienced rater, who was blind to diagnosis and other clinical information, counted the consecutive coronal 0.5-mm slice images in which the CSP was present. The length of the CSP was determined by counting the number of coronal slices showing the CSP. In previous studies, the cut-off length of an abnormal CSP has ranged from 4 mm to 6 mm [13-16]. We adopted a criterion of ≥ 6 mm in CSP length to define an abnormally enlarged CSP (Figure **1**).

Intra- and inter-rater reliabilities for counting coronal image slices with CSP were 0.96 and 0.98, respectively, as assessed by kappa statistics of intraclass correlation coefficients.

### Data Analysis

Continuous variables in demographic and clinical characteristics were compared by independent t-tests, while categorical variables were assessed using χ^2^ tests.

Binary and ordinal logistic regression analyses were used to compare the prevalence of CSP enlargement between groups. Odds ratios (OR) were calculated to estimate risks for opiate dependence. Age and sex compositions were included in each logistic regression model as relevant covariates. Analyses of covariance were performed for the comparison of the continuous CSP length between groups including age and sex as covariates. Associations between onset of opiate use and length of the CSP were examined using the test for linear trends [20].

Statistical significance was defined at the 0.05 level, two-tailed. Stata 11 for Windows was used for all analyses (Stata Corp, College Station, TX, USA).

## Results

### Sample Characteristics

There were no differences in age (t=0.11, p=0.92) and sex composition (χ^2^ =0.03, p=0.87) between all opiate-dependent and healthy subjects (Table **1**). In addition, demographic characteristics in the adolescent-onset (age, t=0.81, p=0.42; sex composition, χ^2^=0.01, p=0.92) and the adult-onset (age, t=-0.96, p=0.34; sex composition, χ^2^=0.04, p=0.85) opiate-dependent groups were not different from those in the healthy comparison group. By definition, adolescent-onset opiate-dependent individuals had earlier ages at first opiate use (t=11.8, p<0.001) and longer durations of illness (t=-2.87, p=0.006) than those whose onsets were during adulthood. There was no significant difference in demographic characteristics (age, t=1.56, p=0.12; sex, Fisher exact p=0.82; ethnicity, χ^2^=1.24, p=0.54; level of education, χ^2^=2.37, p=0.31) and past comorbid substance-related problems on cocaine (χ^2^=1.30, p=0.26) and marijuana (χ^2^=0.33, p=0.57) between subjects with adult-onset opiate dependence and those with adolescent-onset opiate dependence (Table **1**). 

**Table 1:** Demographic and clinical characteristics of study subjects.

	**Opiate-dependent subjects**			
**Characteristics**	**Adolescent-onset group (n=41)**	**Adult-onset group (n=24)**	**All abusers (n=65)**	**Healthy subjects (n=67)**
n	41	24	65	67
Age, mean (SD), y	37.9 (9.2)	41.5 (8.5)	39.2 (9.0)	39.4 (9.4)
Male sex, No. (%)	21 (51.2)	12 (50.0)	33 (50.8)	35 (52.2)
Opiate use				
Age of first use, mean (SD), y	16.4 (2.4)	28.3 (5.6)	20.8 (6.9)	NA
Duration, mean (SD), y	19.9 (9.8)	13.3 (7.3)	17.5 (9.4)	NA
Comorbidity of other illegal substance abuse longer than 1 year				
Cocaine	12 (29.3)	4 (16.7)	16 (24.6)	NA
Marijuana	13 (31.7)	6 (25.0)	19 (29.2)	NA
Addiction severity index *				
Medical	0.32 (0.34)	0.56 (0.36)	0.40 (0.37)	NA
Employment	0.35 (0.35)	0.43 (0.30)	0.38 (0.33)	NA
Alcohol	0.05 (0.15)	0.02 (0.02)	0.04 (0.12)	NA
Drug	0.28 (0.09)	0.28 (0.06)	0.28 (0.08)	NA
Legal	0.17 (0.24)	0.28 (0.27)	0.21 (0.25)	NA
Family	0.19 (0.23)	0.15 (0.25)	0.18 (0.24)	NA
Psychological	0.34 (0.22)	0.41 (0.24)	0.37 (0.23)	NA

* Data from 15 opiate-dependent subjects were not available.

Abbreviations: SD, standard deviation; NA, not available or not applicable

The Addiction Severity Index was administered in the subsample of opiate-dependent subjects (n=50). The results from 7 assessment dimensions are presented in Table **1**. Composite scores of the medical dimension were higher in the adult-onset than in the adolescent-onset opiate-dependent group (t=2.35, p=0.02) while those of other assessment dimensions including current alcohol problems were similar between groups. 

### Risks for Opiate Dependence

Twelve subjects out of 65 opiate-dependent individuals (18.5%) had enlarged CSPs greater than or equal to 6 mm, while 4 healthy comparison subjects (6.0%) did. On binary logistic regression analyses (Table **2**), having an enlarged CSP was associated with a higher likelihood of opiate dependence (adjusted OR=3.64, 95% confidence interval [CI]=1.10-12.04, p=0.034).

**Table 2:** The odds ratios for opiate dependence according to the presence of cavum pellucidum.

	Opiate dependence	Controls	Odds ratio ^a^	95% CI	*p* value
Categorical measures of CSP	No. (%) of subjects				
Presence of CSP	54 (83.1)	43 (64.2)	2.74	1.21 to 6.21	0.016
Presence of large CSP ^b^	12 (18.5)	4 (6.0)	3.64	1.10 to 12.04	0.034
Quantitative measures of CSP	Mean (SD) length of CSP, mm				
Length of CSP	4.17 (5.39)	2.27 (2.10)	1.22	1.05 to 1.42	0.011

^a^Values were risks for opiate dependence in the presence of the CSP and were calculated by the logistic regression model including age and sex as covariates.

^b^A large CSP was defined by the criterion of ≥ 6 mm in CSP length

^c^Values were risks for opiate dependence with each 1-mm increase in CSP length and were calculated by the logistic regression model including age and sex as covariates

Abbreviations: CSP, cavum septum pellucidum; No., number; SD, standard deviation; CI, confidence interval

When the length of the CSP was considered as a continuous variable, the risk of opiate dependence increased by the odds of 1.22 (95% CI=1.05-1.42, p=0.011) with each 1-mm increase in the CSP length. The overall length of the CSP was also greater in opiate-dependent subjects than in healthy subjects (F_1,128_=7.56, p=0.007).

### Risks for Adolescent-Onset Opiate Use

**Figure 2:**
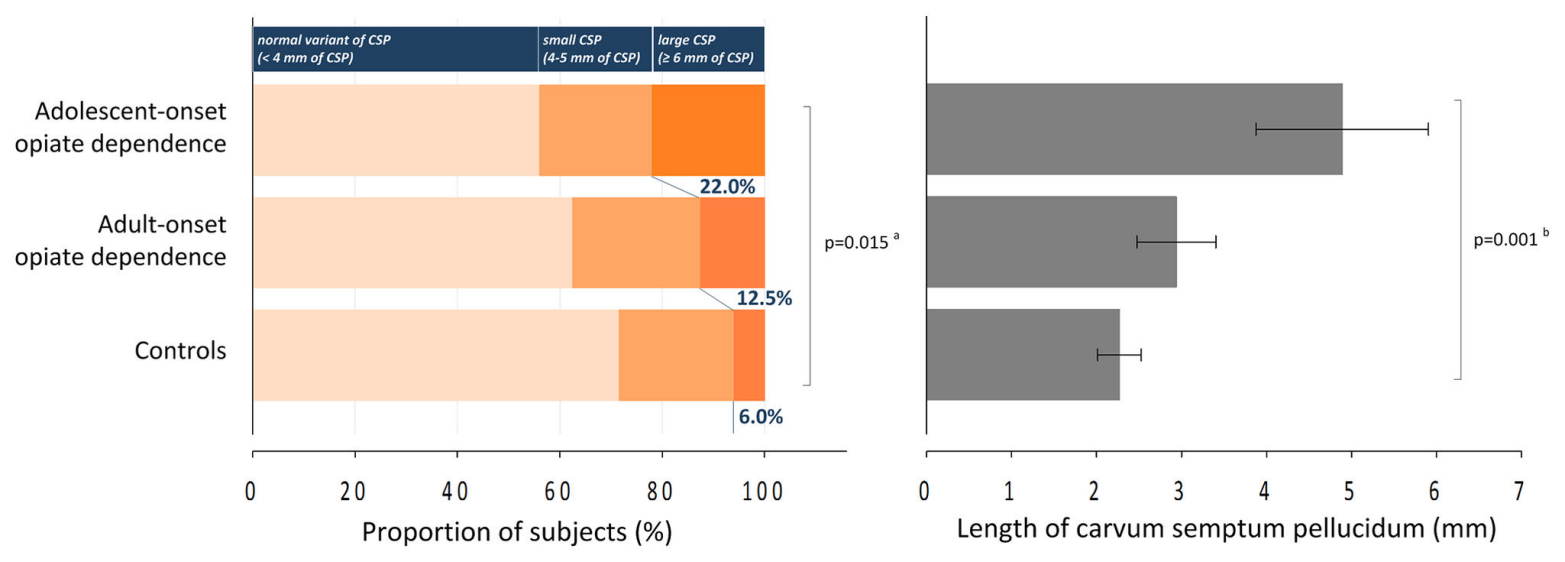
Prevalence of CSP enlargement and mean values of CSP length among adolescent-onset and adult-onset opiate-dependent subjects and healthy subjects. ^a^ p value was calculated by using the ordinal logistic regression model for the comparison of the prevalence of an enlarged CSP (≥ 6 mm in CSP length) among adolescent-onset and adult-onset opiate-dependent subjects and healthy subjects, including age and sex composition as relevant covariates. ^b^ p value was calculated by using the analyses of covariance for linear trend for the comparison of the continuous CSP length between groups including age and sex as covariates. Error bars in the right graph indicate standard errors. Abbreviations: CSP, cavum septum pellucidum.

We also investigated whether having a larger CSP would be associated with an earlier onset of opiate use. Ordinal logistic regression analysis demonstrated a stepwise increase in the prevalence of CSP enlargement in the adult-onset (n=3, 12.5%) and adolescent-onset (n=9, 22.0%) opiate-dependent groups, compared with healthy subjects (adjusted OR=3.58, 95% CI=1.28-9.99, p=0.015)(Figure **2**). One adolescent-onset opiate-dependent subject had an enlarged CSP in cleft shape that may be associated with potential traumatic injury [21,22]. Although a history of head trauma with loss of consciousness was a relevant exclusion criterion in the current study, repeated analysis of the data excluding the subject with cleft-shaped CSP enlargement was performed to ensure the robustness of the findings. Similar results were produced that support a higher risk for adolescent-onset opiate dependence in subjects with CSP enlargement (adjusted OR=3.20, 95% CI=1.13-9.05, p=0.028).

There were also stepwise overall CSP length enlargements between subgroups (control < adult-onset opiate-dependent group < adolescent-onset opiate-dependent group, mean length [SD], 2.27 mm [2.10] < 2.94 mm [2.28] < 4.89 mm [6.49], z=3.43, p for trend =0.001)(Figure **2**). Pairwise comparison of overall CSP length confirmed a larger CSP in opiate-dependent individuals who began drug use in adolescence as compared with adult-onset subjects (adolescent-onset > adult-onset opiate-dependent groups, F_1,61_=4.43, p=0.039). Alcohol-related problems, which also could be associated with CSP enlargement [23], were assessed. Additional covariation of severity of alcohol problems measured using the Addiction Severity Index did not change the results (F_1,45_=4.86, p=0.033).

Similarly, the risk for early adolescent-onset opiate use (adjusted OR=4.72, 95% CI=1.32-16.90, p=0.017) was approximately twice as high as that for adult-onset opiate use (adjusted OR=2.57, 95% CI=0.51-12.95, p=0.25) when CSP enlargement was observed.

## Discussion

The present study provides the first *in vivo* human evidence that neurodevelopmental abnormalities of the limbic structures may be associated with subsequent and earlier development of opiate dependence. We found a greater prevalence of CSP enlargement in opiate-dependent subjects, which potentially represents limbic dysgenesis at early stages of development. As suggested by previous findings, the abundant expression of opioid receptors in the limbic system [24] may also account for the increased likelihood of opiate dependence in individuals with enlarged CSPs. Since other conditions that might elicit CSP abnormality, such as major comorbid psychiatric disorders with neurodevelopmental origin [13,16], were excluded from the study, it is unlikely that these potential confounding conditions had contributed to the present results.

It is notable that an abnormally enlarged CSP was associated with an earlier onset of illicit drug use, especially an onset during adolescence. The present findings suggest that illicit drug-seeking behaviors of adolescents may have a strong neurodevelopmental disposition, although it is not clear whether the pathophysiology of adolescent-onset addiction may be etiologically different from that of adult-onset addiction. The prefrontal-limbic circuit, which is implicated in motivation, inhibition, emotion, and judgment, undergoes maturation throughout adolescence to reach its full potential in adulthood [6,25]. Accordingly, constitutional abnormalities in the limbic system may render the adolescent brain more prone to impaired decision-making regarding substance use, thereby precipitating transition from experimental drug-taking to full-blown addiction.

The current findings are broadly in agreement with the previous literature, which suggests that brain structural deficits, particularly in the amygdala, may be an antecedent event that could predispose to subsequent substance abuse [9]. It is also notable that CSP enlargement has been associated with antisocial personality characteristics, which are closely related to risky behaviors and limited appreciation of consequences [7].

Although the functional relevance of abnormal CSP enlargement has not been fully explored to date, convergent evidence suggests that an enlarged CSP appears to be related to prenatal abnormalities of neighboring structures including the corpus callosum, hippocampus, amygdala, and septal nuclei [10-12]. A handful of neuroimaging studies have reported that individuals with enlarged CSPs show volume reductions in these major limbic structures [26-28]. The current findings may thus indicate pre-existing abnormalities of brain regions neighboring the CSP in opiate-dependent individuals.

Among the limbic structures, the septal area has long received attention in addiction research [29]. Decades ago, experiments involving rats with electrodes implanted in the septal area of the basal forebrain provided insight on the importance of this brain region in mediating the experience of reward and pleasure [30]. Since then, a substantial amount of preclinical and clinical evidence has suggested that the septal nuclei encompassed by the septum pellucidum have primarily been implicated in drug sensitization and reinforcement, along with other major limbic structures including the nucleus accumbens, amygdala, hippocampus, and thalamus [29]. The septal area is a major triggering brain region for reward behaviors [30,31], and lesions of this brain area may enhance drug sensitization [32]. From the perspective that hyper-sensitization to drugs or drug-related stimuli may, in part, account for tendency toward addiction [33], early maldevelopment of the septal area and subsequent CSP enlargement may potentially render affected individuals hyper-salient to effects of drugs. The present findings may bolster this hypothesis as they indicate that abnormal CSP enlargement confers an increased risk of earlier onset of illicit opiate use.

Thus far, CSP enlargement has most frequently been studied in schizophrenia spectrum disorders wherein the neurodevelopmental aberration is etiologically significant [17]. Intriguingly, we found that the prevalence of CSP abnormality in opiate-dependent subjects (18.5%) appears to be much higher than that observed in persons with schizophrenia spectrum disorders, as reported in a recent meta-analysis (8.1%)[17]. Considering that the frequency of CSP enlargement in healthy subjects of this cohort (5.97%) is similar to that observed in the meta-analysis (4.50%), it might be assumed that the effects of abnormal CSP on the risk for opiate dependence (OR=3.64) could be larger than those on the risk for schizophrenia spectrum disorders (OR=1.59). Since the previous meta-analysis included all available studies on CSP [17], we selectively searched and mini-reviewed recent literature focusing on the homogenous sample of schizophrenia and affective disorders using the same criterion of CSP enlargement (≥ 6mm) as in our study (Table S1 and Figure S1 in File **S1**). This selective review also demonstrated the significant effect of CSP enlargement on the development of schizophrenia (OR=2.20, 95% CI=1.43-3.40) while the relationship between CSP abnormality and affective disorder were controversial (OR=1.30, 95% CI=0.66-2.58). However, an increased prevalence of CSP enlargement was observed in the subgroup of bipolar patients with earlier onset [13]. Even when compared to the results from this review, the predictability of the presence of CSP enlargement for adolescent-onset opiate dependence (OR=4.72) also appears to be greater than that for schizophrenia. Maldevelopment of the septal nuclei, which directly influence drug reward and sensitization, may contribute to this larger effect of CSP enlargement on the development of substance dependence. More comprehensive imaging and postmortem studies are warranted to determine the role of the developmental disturbances within the septal area in later substance dependence.

Several heritable and perinatal environmental contributions have been suggested to cause incomplete fusion of the leaves of the septum pellucidum and following CSP abnormality [34]. Tentative heritable contributions to the occurrence of CSP abnormality have recently been reported [35], and prenatal insults including alcohol exposures, smoking, or infections may also contribute to limbic maldevelopment related to CSP enlargement [36]. The current findings suggest that disruption in early brain development during prenatal and very early postnatal periods may lead to abnormal CSP enlargement that can have lasting influences potentially contributing to early involvement in addictive behaviors.

There are several limitations of the present study that should be taken into consideration in interpreting the results. As the focus of the study was on CSP enlargement in opiate dependence, it did not delve much into the issue of whether this limbic abnormality would be a common neurodevelopmental mechanism shared by a broad spectrum of addictive behaviors. Future studies that include subjects with other types of substance and behavioral addictive disorders would be necessary to further explore the issue.

Furthermore, even though the current findings strongly support the neurodevelopmental roles of the limbic structures in the pathophysiology of opiate dependence, interactions with environmental and genetic factors combined may affect vulnerability. In a related limitation, repeated head injuries have been known to contribute to an acquired form of CSP enlargement, which appears to have a different morphological shape from CSP abnormality of neurodevelopmental origin [21-23]. A cleft-shape CSP with a tear in septal leaflets might be the most robust form of CSP enlargement elicited by head trauma [21,22] while a longer and wider shaped CSP suggests a CSP enlargement of neurodevelopmental origin [23,37]. In an effort to avoid possible confounding effects of CSP enlargement induced by head trauma, we tried to exclude subjects with a history of significant head trauma in the present study. Furthermore, repeated analysis excluding cleft-shaped CSP enlargement, which could be potentially related to head trauma, was also performed. 

Additionally, alcohol-related problems might be another contributing factor to CSP enlargement [23]. Although the severity of alcohol problems did not influence the results, future studies would be required in larger cohorts including both disease entities.

Without a group of adolescents with exposure to opiates who do not develop chronic addiction problems, the current cross-sectional study design could not determine whether abnormal CSP enlargement is associated with early, during adolescence, opiate use initiation, regardless of prolonged addiction problems or early-onset chronic opiate use. A recent longitudinal study that examined 30-year trajectories of heroin and other drug use among young adults with heroin dependence has suggested associations between antisocial personality traits and persistent heroin use at a younger age [38]. 

Future longitudinal studies including a group of adolescents with exposure to illicit drug who do not develop chronic addiction problems would be necessary to determine whether abnormal CSP enlargement would be a potential neurobiological marker for only the earlier exposure to opiate or for the prolonged opiate use problems after initial exposure.

In addition, there is a possibility that some psychosocial factors also mediate a link between abnormal CSP enlargement and adolescent-onset opiate use. In the current study, however, sociodemographic characteristics were similar between adult-onset and adolescent-onset opiate-dependent subjects. Given the links between CSP enlargement and antisocial personality characteristics [7] and between antisocial personality characteristics and persistent opiate use [38], it is recommended to included antisocial personality traits in future studies. Future longitudinal studies with more detailed sociodemographic characteristics and larger sample size may help find the potential psychosocial factors that indirectly mediate the link between abnormal CSP enlargement and adolescent-onset opiate use.

In conclusion, the current findings suggest that the presence of abnormal CSP enlargement might be considered as a neurodevelopmental marker or a trait that increases the risk of earlier exposure to illicit drugs including opiate. Namely, adolescent-onset opiate dependence might have more neurodevelopmentally determined predisposition in its etiology, as compared with adult-onset dependence. Given the challenging issue of which brain regional disruptions might predict the population more prone to later addiction [8], the present findings may have predictive relevance for vulnerability to early exposure to highly addictive substances. Identification of this developmental marker among higher-risk individuals, particularly at an earlier stage of substance use, would contribute to appropriate prevention and treatment for the vulnerable population. Such a marker could also facilitate prospective studies in adolescents who may be at increased risk for developing opioid dependence.

## Supporting Information

File S1 **Supporting information.** Table S1, Recent literature on the prevalence of cavum septum pellucidum enlargement in schizophrenia or affective disorders. Figure S1, The odds ratios of recent magnetic resonance imaging studies that assessed the prevalence of cavum septum pellucidum enlargement (≥ 6mm) in cohorts of schizophrenia or affective disorders.
(DOCX)
