# Supplementary material for: Enlarged Cavum Septum Pellucidum as a Neurodevelopmental Marker in Adolescent-Onset Opiate Dependence
Source: PLoS One. 2013 Oct 24;8(10):e78590. doi: 10.1371/journal.pone.0078590 (PMC3813473; doi:10.1371/journal.pone.0078590)
Supplement: File S1 — Supporting information. Table S1, Recent literature on the prevalence of cavum septum pellucidum enlargement in schizophrenia or affective disorders. Figure S1, The odds ratios of recent magnetic resonance imaging studies that assessed the prevalence of cavum septum pellucidum enlargement (≥ 6mm) in cohorts of schizophrenia or affective disorders. (DOCX) [file pone.0078590.s001.docx]

**Enlarged cavum septum pellucidum as a neurodevelopmental marker in adolescent-onset opiate dependence**

***Supporting Information (File S1)***

**Table S1.** Recent literature on the prevalence of cavum septum pellucidum enlargement in schizophrenia or affective disorders ^a^

**Figure S1.** The odds ratios of recent magnetic resonance imaging studies that assessed the prevalence of cavum septum pellucidum enlargement (≥ 6mm) in cohorts of schizophrenia or affective disorders ^a^

| **Supplemental Table 1.** Recent literature on the prevalence of cavum septum pellucidum enlargement in schizophrenia or affective disorders ^a^ | | | |
| --- | --- | --- | --- |
| **Studies** | **Events / Total number of subjects** | | **Odds ratio (95% CI)** |
|  | **Disease** | **Control** |  |
| ***Schizophrenia*** |  |  |  |
| Nopoulos et al (1997) ^1^ | 6 / 55 | 1/ 75 | 9.06 (1.06 - 77.60) |
| Nopoulos et al (1998) ^2^ | 4 / 24 | 3 / 95 | 6.13 (1.27 - 29.57) |
| Kwon et al (1998) ^3^ | 7 / 30 | 4 / 46 | 3.20 (0.85 - 12.08) |
| Hagino et al (2001) ^4^ | 6 / 86 | 3 / 79 | 1.90 (0.46 - 7.87) |
| Rajarethinam et al (2001) ^5^ | 3 / 73 | 1 / 43 | 1.80 (0.18 - 17.87) |
| Kasai et al (2004) ^6^ | 6 / 33 | 4 / 56 | 2.89 (0.75 - 11.12) |
| de Souza Crippa et al (2006) ^7^ | 8 / 38 | 1 / 38 | 9.87 (1.17 - 83.35) |
| Flashman et al (2007) ^8^ | 11 / 77 | 5 / 55 | 1.67 (0.54 - 5.10) |
| Takahashi et al (2008) ^9^ | 19 / 192 | 10 / 87 | 0.85 (0.38 -1.90) |
| Total | 70 / 608 | 32 / 574 | 2.20 (1.43 -3.40) |
| ***Affective disorders*** |  |  |  |
| Kwon et al (1998) ^3^ | 2 / 21 | 4 / 46 | 1.11 (0.19 - 6.57) |
| Kasai et al (2004) ^6^ | 6 / 41 | 4 / 56 | 2.23 (0.59 - 8.48) |
| Kim et al (2007) ^10^ | 8 / 41 | 1 / 41 | 9.70 (1.15 - 81.55) |
| Takahashi et al (2009) ^11^ | 3 / 56 | 5 / 33 | 0.32 (0.07 - 1.42) |
| Takahashi et al (2010) ^12^ | 1 / 26 | 3 / 24 | 0.28 (0.03 - 2.90) |
| Total | 20 / 185 | 17 / 200 | 1.13 (0.66 - 2.58) |

^a^ Studies using the criterion of cavum septum pellucidum enlargement (≥ 6mm) were included in the review.

| **Supplementary Figure 1.** The odds ratios of recent magnetic resonance imaging studies that assessed the prevalence of cavum septum pellucidum enlargement (≥ 6mm) in cohorts of schizophrenia or affective disorders ^a^ |
| --- |
| __ |
| ^a^ In this selective mini-review, we only included studies that focused on the homogenous sample of schizophrenia or affective disorders using the same criterion of cavum septum pellucidum enlargement (≥ 6mm) as in our study.  The mean data represent the significantly high prevalence of abnormal cavum septum pellucidum enlargement in schizophrenia but not in affective disorders. Note that the risk for adolescent-onset opiate dependence when having an enlarged CSP (OR=4.72, 95% CI=1.32-16.90) appears to be approximately twice as high as that for schizophrenia. |

**References**

1. Nopoulos P, Swayze V, Flaum M, Ehrhardt JC, Yuh WT, Andreasen NC: Cavum septi pellucidi in normals and patients with schizophrenia as detected by magnetic resonance imaging. Biol Psychiatry 1997; 41:1102-1108

2. Nopoulos PC, Giedd JN, Andreasen NC, Rapoport JL: Frequency and severity of enlarged cavum septi pellucidi in childhood-onset schizophrenia. Am J Psychiatry 1998; 155:1074-1079

3. Kwon JS, Shenton ME, Hirayasu Y, Salisbury DF, Fischer IA, Dickey CC, Yurgelun-Todd D, Tohen M, Kikinis R, Jolesz FA, McCarley RW: MRI study of cavum septi pellucidi in schizophrenia, affective disorder, and schizotypal personality disorder. Am J Psychiatry 1998; 155:509-515

4. Hagino H, Suzuki M, Kurokawa K, Mori K, Nohara S, Takahashi T, Yamashita I, Yotsutsuji T, Kurachi M, Seto H: Magnetic resonance imaging study of the cavum septi pellucidi in patients with schizophrenia. Am J Psychiatry 2001; 158:1717-1719

5. Rajarethinam R, Miedler J, DeQuardo J, Smet CI, Brunberg J, Kirbat R, Tandon R: Prevalence of cavum septum pellucidum in schizophrenia studied with MRI. Schizophr Res 2001; 48:201-205

6. Kasai K, McCarley RW, Salisbury DF, Onitsuka T, Demeo S, Yurgelun-Todd D, Kikinis R, Jolesz FA, Shenton ME: Cavum septi pellucidi in first-episode schizophrenia and first-episode affective psychosis: an MRI study. Schizophr Res 2004; 71:65-76

7. de Souza Crippa JA, Zuardi AW, Busatto GF, Sanches RF, Santos AC, Araujo D, Amaro E, Hallak JE, Ng V, McGuire PK: Cavum septum pellucidum and adhesio interthalamica in schizophrenia: an MRI study. Eur Psychiatry 2006; 21:291-299

8. Flashman LA, Roth RM, Pixley HS, Cleavinger HB, McAllister TW, Vidaver R, Saykin AJ: Cavum septum pellucidum in schizophrenia: clinical and neuropsychological correlates. Psychiatry Res 2007; 154:147-155

9. Takahashi T, Yung AR, Yucel M, Wood SJ, Phillips LJ, Harding IH, Soulsby B, McGorry PD, Suzuki M, Velakoulis D, Pantelis C: Prevalence of large cavum septi pellucidi in ultra high-risk individuals and patients with psychotic disorders. Schizophr Res 2008; 105:236-244

10. Kim MJ, Lyoo IK, Dager SR, Friedman SD, Chey J, Hwang J, Lee YJ, Dunner DL, Renshaw PF: The occurrence of cavum septi pellucidi enlargement is increased in bipolar disorder patients. Bipolar Disord 2007; 9:274-280

11. Takahashi T, Yucel M, Lorenzetti V, Nakamura K, Whittle S, Walterfang M, Suzuki M, Pantelis C, Allen NB: Midline brain structures in patients with current and remitted major depression. Prog Neuropsychopharmacol Biol Psychiatry 2009; 33:1058-1063

12. Takahashi T, Malhi GS, Wood SJ, Yucel M, Walterfang M, Nakamura K, Suzuki M, Pantelis C: Midline brain abnormalities in established bipolar affective disorder. J Affect Disord 2010; 122:301-305
